# Efficacy and Safety of Acupoint Catgut Embedding for Diarrhea-Predominant Irritable Bowel Syndrome and Constipation-Predominant Irritable Bowel Syndrome: A Systematic Review and Meta-Analysis

**DOI:** 10.1155/2020/5812320

**Published:** 2020-11-27

**Authors:** Jing Wu, Qinwei Fu, Shasha Yang, Hui Wang, Yaofeng Li

**Affiliations:** ^1^Guizhou University of Traditional Chinese Medicine, Guiyang 550025, China; ^2^Hospital of Chengdu University of Traditional Chinese Medicine, Chengdu University of Traditional Chinese Medicine, Chengdu 610072, China; ^3^The First Affiliated Hospital of Guizhou University of Traditional Chinese Medicine, Guiyang 550002, China

## Abstract

In this study, we aim to evaluate the efficacy and safety of acupoint catgut embedding for the treatment of diarrhea-predominant irritable bowel syndrome and constipation-predominant irritable bowel syndrome. We searched seven online databases to collect studies published up to Feb 29^th^, 2020. Study quality of each included article was evaluated by the Cochrane Collaboration Risk of Bias Tool. Systematic reviews and meta-analyses were conducted based on the Cochrane systematic review method by using RevMan 5.3 software. Among the included trials, acupoint catgut embedding alone or plus oral western medicine or plus other acupoint-based therapies, or plus oral traditional Chinese medicine were the main therapies in the experimental groups. Interventions in control groups mainly include oral western medicine alone, other acupoint-based therapies alone, or other acupoint-based therapies alone. Primary outcomes in this study include recovery rate, accumulative marked effective rate, accumulative effective rate, and recurrence rate. Finally, 30 trials involving 1889 participants were included. The results of systematic reviews and meta-analyses show that acupoint catgut embedding alone or plus oral western medicine or plus other acupoint-based therapy or plus oral traditional Chinese medicine was significantly better compared with using oral western medicine alone in terms of efficacy for IBS-C and IBS-D. In addition, acupoint catgut embedding alone or plus oral western medicine or plus other acupoint-based therapy or plus oral traditional Chinese medicine could improve the effective rate and decrease the recurrence rate for IBS-D compared with using oral western medicine, other acupoint-based therapies, or oral traditional Chinese medicine alone. Adverse events of acupoint catgut embedding include local induration, redness, swelling, and itchiness, but they were all mild and disappeared swiftly with ordinary local interventions. There is an urgent need for RCTs of high quality and large sample size and with longer treatment duration and follow-up periods of acupoint catgut embedding for IBS.

## 1. Introduction

Considered the most common functional gastrointestinal disorder worldwide, irritable bowel syndrome (IBS) is characterized by chronic and recurrent abdominal pain and (or) altered bowel habits, which could not be explained by any anatomical or structural abnormality [1, 2].

According to Rome IV criteria, IBS is divided into four subtypes in view of symptoms, including diarrhea-predominant IBS (IBS-D), constipation-predominant IBS (IBS-C), IBS with mixed symptoms of diarrhea and constipation (IBS-M), and untyped IBS (IBS-U) [1]. Gastrointestinal tract motility, gut microbiotic imbalance, and visceral hypersensitivity contribute to IBS mainly, while more researches on its mechanisms are still needed urgently [3].

As of 2014, more than 23% of the world population was suffering from IBS with decreased work productivity and life quality [4]. The international overall medical costs concerning IBS were more than 200 billion dollars in 2008, with potential indirect burden [5]. Conventional drugs, such as fiber supplementation, antispasmodics, probiotics, antidepressants, and psychological treatments, are used to alleviate the symptoms, but the effects are limited and followed by various adverse events [6–8].

Evidence from some RCTs favored several traditional Chinese medicine (TCM) therapies for IBS, such as TCM herbal decoctions, acupuncture, and moxibustion, with a considerable efficacy, lower recurrence rate, and fewer adverse events [9–11]. As a kind of TCM external therapy, acupoint catgut embedding (ACE) was a combination of TCM acupuncture theory (based on meridians and acupoints) and modern technologies (catgut embedding with special syringe), which is being applied widely especially in China. In addition, it is easier to operate and has more durable stimulation compared with acupuncture [12]. However, the use of ACE in the treatment of IBS beyond China is not popular, and the clinical efficacy of ACE alone or with other therapies is not certain.

Several reviews concerning TCM internal (TCM herbal medicine decoctions) and external (acupuncture and moxibustion) therapies for IBS have been published [13–20], while no study on the efficacy and safety of ACE for IBS has been conducted. The aim of our study is to identify the clinical efficacy and safety of ACE for the treatment of IBS-C and IBS-D and to compare the efficacy and adverse effects of applying OWM, OTCM, or other ABT alone by several comparisons.

## 2. Material and Methods

### 2.1. Protocol and Registration

This systematic review was registered in PROSPERO, an international prospective register of systematic reviews, with the registration number CRD42020163031 (available from https://www.crd.york.ac.uk/prospero/display_record.php?RecordID=163031).

### 2.2. Search Strategy

We searched seven electronic databases, including Embase, PubMed, Cochrane Library, the China National Knowledge Infrastructure (CNKI), Technology Periodical Database (VIP), Wanfang Data Information Site, and SinoMed (CBM) up to Feb 29^th^, 2020. The search strategy and inclusion criteria were decided according to the guidance of the PRISMA agreement. We used the following two groups of search terms in English: (1) “irritable bowel syndrome,” “IBS” connected with “OR”; (2) “acupoint catgut embedding,” “catgut embedding therapy,” “catgut implantation at acupoint,” “point embedding therapy” connected with “OR.” The previously mentioned search terms of (1) and (2) were connected with term “AND.” All searches were limited to trials of RCT in humans and were conducted in electronic databases by two authors independently. We also searched with related search terms in Chinese and searched the references of the original and review articles manually for possible related trials and also tried to get grey literatures identified through other sources.

### 2.3. Inclusion Criteria

In this systematic review, we searched and included trials according to the following criteria:

1. Trials with participants that were diagnosed with IBS-D or IBS-C according to certain guidelines were included.
2. Prospective randomized controlled trials (RCTs) were included.
3. Trials in which acupoint catgut embedding alone or plus other therapy(ies) were applied in experimental groups. The patients in control groups received conventional therapy(ies), other TCM therapies, or placebo regimens. Trials for IBS-D or IBS-C combined with other disease(s) or for IBS but without classification of certain type (IBS-D or IBS-C) were excluded.
4. Primary outcomes included recovery rate, accumulative marked effective rate, accumulative effective rate, and recurrence rate.
5. Trials in Chinese or English were included.

### 2.4. Study Selection and Data Extraction

According to the aforementioned design, two reviewers (Jing Wu and Qinwei Fu) searched the online databases listed previously and recorded the titles and abstracts of all the articles. Two evaluators (Hui Wang and Yaofeng Li) assessed the eligibility of these articles and made decisions on every research (inclusion or exclusion) independently. If they did not reach the same decision, the concerned articles were discussed with a fourth reviewer (Shasha Yang). Two reviewers (Jing Wu and Qinwei Fu) extracted the data independently from each study. Differences in the extracted data were solved after discussion with a fourth reviewer (Shasha Yang).

### 2.5. Quality Assessment

Quality assessment of all the trials included in this review was independently evaluated by three reviewers (Jing Wu, Hui Wang, and Qinwei Fu) using the Cochrane Collaboration Risk of Bias Tool by RevMan 5.3 software. Any disagreement was resolved by discussions with a fourth reviewer (Shasha Yang).

### 2.6. Statistical Analysis

This systematic review and meta-analysis was performed with the RevMan 5.3 software. Recovery rate, accumulative marked effective rate, accumulative effective rate, and recurrence rate were considered as dichotomous data, and some findings such as abdominal pain score, abdominal distention score, anorectal resting pressure, rectal maximum tolerance capacity, defecation frequency score, and mucinous stool score were considered as continuous data. Risk ratio (RR) and mean difference (MD) with 95% confidence intervals (CIs) were given separately, which was an estimate of the pooled effect sizes, and *P* values of less than 0.05 were considered statistically significant.

For the assessment of heterogeneity, we evaluated trials using both the *I*^2^ statistic and Chi-square test (*P* < 0.1), which indicates the proportion of variability across trials not explained by sampling variation alone, and the *P* value of the *V*^2^ test of heterogeneity.

*P* < 0.1 or *I*^2^ > 50% indicated significant heterogeneity. If significant heterogeneity was not observed, a fixed-effects model was used to make estimates; otherwise, a random-effects model was applied to statistical analysis.

Exploration of publication bias was planned if more than ten trials were included. Due to the number of included trials and methodological quality, not all planned analyses could be available.

## 3. Results

### 3.1. Study Inclusion

Initially, 293 records were searched from seven databases with no grey literature reference. After the removal of duplicates, the records were decreased to 87. Based on the titles and abstracts of records, we excluded 31 papers with reasons such as observational studies, case reports, uncontrolled studies, animal experiments, reviews, and studies with no randomization-control design and not related to acupoint catgut embedding for IBS-D or IBS-C. The remaining 56 articles were downloaded for further selection, and 30 articles were excluded with reasons (Figure S1). Eventually, 30 trials form 26 studies (one four-arm study was recombined to five trials for comparison) were included [21–46]. The flow diagram of the study selection process is shown in Figure S1.

### 3.2. Study Characteristics

All 30 included RCTs were conducted in China and published in Chinese, with the range of publish years from 2008 to 2019 [21–46]. In total, 699 participants (11 trials) aging from 16 to 75 with IBS-C from three months to 12 years and 1190 participants (19 trials) aging from 18 to 69 with IBS-D from 15 days to 22 years were involved. Details of baseline characteristics were not reported in several trials, but no significant difference between groups (*P* < 0.05) among the characteristics was mentioned in all of them. As for the interventions of experimental groups, ACE alone, or combined with OTCM (TCM decoction/powder/granule) or with other ABT (e.g., auricular therapy, acupressure therapy for IBS-C, and fire needle therapy, moxibustion, and acupoint application for IBS-D) or with OWM (same as the medicine used in the control groups mostly), was applied in both IBS-C and IBS-D. In addition, ACE plus *Tai Chi Chuan* or diet therapy was applied in two trials (IBS-C) separately [36, 43]. Though some of the specific prescriptions of the TCM decoction/powder/granule applied were different among the included trials, their principles and theories showed similarity according to the theory of TCM. In addition, OWM alone was applied in control groups of 10 trials for IBS-C [21, 36–42, 44–46], with OWM plus sham ACE in one trial [43]. As for the control groups for IBS-D, OWM alone was applied in 11 trials [22, 24, 25, 27, 29, 31–35], TCM decoction/powder in five trials [23, 26, 28, 33], other ABT (acupuncture and acupoint application) in two trials [21, 30], and ACE plus *Buzhong Yiqi* decoction (TCM) in one trial [33]. Detailed characteristics of the included trials are listed in Tables S1 and S2.

### 3.3. Assessment of Quality and Bias

**Figure 1:**
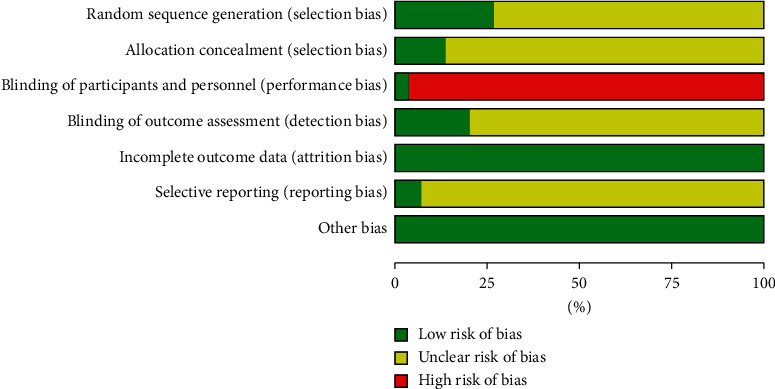
Risk of bias graph.

**Figure 2:**
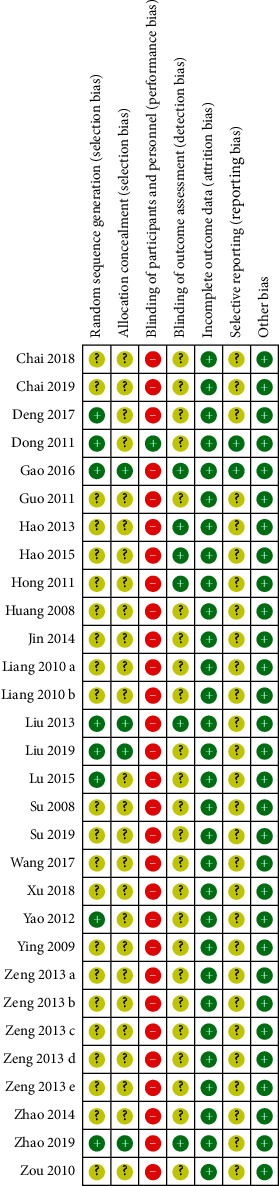
Risk of bias summary.

According to the results of Cochrane Collaboration Risk of Bias Tool [47], eight trials described the method of randomization clearly and appropriately with no trial in high risk of bias [22, 26, 27, 31, 35, 37, 38, 43]. The method of allocation concealment was described clearly in four trials [26, 35, 37, 38] but was unclear in the others. No trial reported blinding method in addition to one study with sham ACE applied in the control groups [43]. Specially assigned procedures with blinding for outcome assessment were applied in six trials [24, 26, 35, 38, 42, 46], and two trials reported protocol or registration ahead of experiment [38, 43]. The details are shown in Figures 1 and 2.

### 3.4. Efficacy of ACE in AR Patients

#### 3.4.1. ACE Alone versus OWM Alone (for IBS-C and IBS-D)

Studies included favored ACE alone for both IBS-C [37, 44] and IBS-D [24, 25, 33, 34] on higher recovery rate, accumulative marked effective rate, and accumulative effective rate significantly (*P* < 0.05), with none to mild heterogeneity (*I*^2^: 0%–8%) (Tables 1 and 2; Figures S2 and S4). More and significant (*P* < 0.05) reductions on abdominal pain score (MD = −0.53/−0.75) were reported in ACE alone groups for both IBS-C [37, 44] and IBS-D [25, 33], but with considerable heterogeneity (*I*^2^: 58%–76%) (Figures S3 and 5).

In addition, evidence shows that ACE alone could better relieve abdominal distention for IBS-C patients (MD = −0.26, *P* < 0.05) with no heterogeneity [37, 44] and decrease recurrence rate after three months (MD = 0.49, *P* for MD > 0.05, *I*^2^ = 60%) and defecation frequency (MD = −0.93, *P* for MD > 0.05, *I*^2^ = 99%) for patients with IBS-D compared with the control groups [25, 33] (Figures S2 and S5). However, results show that ACE alone could not improve mucinous stool better compared with applying WM alone (MD = 0.13, *P* for MD > 0.05) for patients with IBS-D with mild heterogeneity (*I*^2^ = 15%) [25, 33] (Figure S5).

#### 3.4.2. ACE plus Other ABT versus OWM Alone (for IBS-C and IBS-D)

With regard to ACE plus other ABT groups compared with OWM alone groups, evidence favored higher accumulative marked effective rate (IBS-C: RR = 1.44; *P* for RR > 0.05; 95% CI: 0.82–2.51; *I*^2^ = not applicable; IBS-D: RR = 1.8; *P* for RR < 0.01; 95% CI: 1.23–2.62; *I*^2^ = 0%) and accumulative effective rate (IBS-C: RR = 1.27; *P* for RR < 0.01; 95% CI: 1.08–1.49; *I*^2^ = 0%; IBS-D: RR = 1.33; *P* for RR < 0.01; 95% CI: 1.09–1.62; *I*^2^ = 35%) for patients with IBS-C [38, 40, 45] or IBS-D [29, 32], respectively. Results also show that ACE plus other ABT could provide significantly higher recovery rate for patients with IBS-C (RR = 2.73; *P* for RR = 0.01; 95%CI: 1.22–6.09; *I*^2^ = 0%) [38, 40, 45] (Tables 1 and 2; Figures S6 and S7).

#### 3.4.3. ACE plus OTCM versus OWM Alone (for IBS-C and IBS-D)

Findings of meta-analysis show that ACE plus OTCM could relieve constipation symptoms better for IBS-C patients compared with OWM alone, including lower anorectal resting pressure (MD = −2.81; *P* for MD = 0.03; 95%CI: −5.32 to −0.3; *I*^2^ = 0%) and rectal maximum tolerance capacity (MD = −19.3; *P* for MD < 0.01; 95%CI: −32.56 to −6.04; *I*^2^ = 77%) [41, 46] (Table 1; Figure S8).

In addition, by comparisons of ACE plus OTCM versus OWM alone, the pooled results favored the experimental groups on recovery rate (RR = 3.36; *P* for RR = 0.29; 95%CI: 0.35–31.93; *I*^2^ = 77%), accumulative marked improvement rate (RR = 1.87; *P* for RR < 0.01; 95%CI: 1.24–2.81; *I*^2^ = 47%), and accumulative effective rate (RR = 1.31; *P* for RR < 0.01; 95% CI: 1.15–1.5; *I*^2^ = 0%) in three trials for IBS-D [22, 27, 33] (Table 2; Figure S9).

#### 3.4.4. ACE plus OTCM versus OTCM Alone (for IBS-D Only)

Results of systematic review demonstrate that ACE plus OTCM could provide better improvements than OTCM alone without heterogeneity for patients with IBS-D (Table 2), including recovery rate (RR = 1.82; *P* for RR = 0.01; 95% CI: 1.14–2.92) [23, 33], accumulative marked improvement rate (RR = 1.31; *P* for RR = 0.07; 95% CI: 0.98–1.74) [28, 33] accumulative effective rate (RR = 1.11; *P* for RR = 0.07; 95% CI: 0.99–1.24) [23, 28, 33], and recurrence rate (6 months) (RR = 0.65; *P* for RR = 0.2; 95% CI: 0.33–1.26) [23, 33] (Figure S10).

#### 3.4.5. ACE plus Other ABT versus Other ABT Alone (for IBS-D Only)

Compared with the other ABT groups, significant improvements were found in ACE plus other ABT groups (Table 2), including accumulative marked effective rate (RR = 1.32; *P* for RR < 0.01; 95% CI: 1.12–1.56; *I*^2^ = 0%) and accumulative effective rate in two trials (RR = 1.23; *P* for RR < 0.01; 95% CI: 1.11–1.35; *I*^2^ = 0%) [21, 30] (Figure S11).

#### 3.4.6. ACE plus OWM versus OWM Alone (for IBS-D Only)

Compared with the control groups, pooled results of two trials show favored ACE plus OWM concerning recovery rate (RR = 2.72; *P* for RR = 0.12; 95% CI: 0.76–9.69; *I*^2^: not applicable), accumulative marked improvement rate (RR = 5.09; *P* for RR = 0.28; 95% CI: 0.27–94.99; *I*^2^ = 78%), and accumulative effective rate (RR = 1.46; *P* for RR < 0.01; 95% CI: 1.21–1.77; *I*^2^ = 0%) [31, 35] (Table 2; Figure S12).

### 3.5. Adverse Events Reported in Trials

Adverse events were only reported for the experimental groups of two trials [26, 35], with no adverse event reported in seven trials (neither in experimental groups nor in control groups). One trial reported local induration on the acupoints (3 cases, 5.08%) after ACE, which was relieved after local normal acupuncture and hot compress [26]. Local redness, swelling, and itchiness for ACE were found in one trial, and the events disappeared after two weeks by local application of iodophor (tid) [35].

Adverse events were not reported in the other 21 trials.

## 4. Discussion

IBS is a chronic functional disorder with clinical severity, which varies from episodic mild pain up to severe daily symptoms [48]. There is no therapy with universal recognition at present, while an increasing number of patients and medical staff have turned to some complementary and alternative medicine therapies for treatment. Some evidence has proved the efficacy and safety of ACE for abdominal obesity, hypertension, diabetes and its chronic complications, postmenopausal osteoporosis, infertility, and allergic rhinitis [49–54]; however, there was no systematic review and meta-analysis of efficacy and safety for IBS. Our systematic review and meta-analysis firstly evaluated efficacy and safety of ACE for both IBS-C and IBS-D, respectively, for more general and comprehensive evidence.

ACE shares many similarities with TCM acupuncture, including theories of TCM, meridian especially, acupoint selection based on syndrome differentiation, and some operation requirements for acupoint-based therapies (e.g., *De Qi* in TCM) [55]. In addition, there exist some differences in techniques and tools (catgut embedding by special syringe at the surface of the body, muscle layer usually, but not by acupuncture needle) and tendency of acupoint selection (acupoints where fat muscle exists, such as waist, back and abdomen which were more preferred, although ACE will be applied for some patients with intractable facial paralysis). Acupoints for acupuncture could be referred for acupoint selection process of ACE on the basis of this.

Results of our study indicate that, compared with OWM alone, pooled results of our study covering 20 trials with 1252 participants favored ACE alone (or plus other ABT or plus OTCM) concerning higher efficacy and lower recurrence rate (ACE alone vs. OWM alone for IBS-C) for IBS-C and IBS-D. In addition, ACE plus OTCM (or plus other ABT or plus OWM) exhibited favorable improvements compared with the control groups (OTCM, other ABT, or OWM alone correspondingly) for IBS-D.

In TCM theory, IBS-C and IBS-D are categorized as constipation disease and diarrhea disease, respectively, which is combined with abdominal pain disease sometimes. Syndromes of IBS fall into excess syndrome (liver depression and *Qi* stagnation, stagnant heat of intestine, damp-heat in spleen and stomach), deficiency syndrome (spleen-kidney *yang* deficiency), and a combination of both (spleen deficiency with damp encumbrance, liver depression, and spleen deficiency) [56]. According to TCM theory, effects of ACE include balancing *yin*, *yang,* and *zang-fu* organs, promoting meridian *Qi*, regulating *Qi* and blood, tonifying for the deficiency and reducing for the excess, strengthening the antipathogenic *Qi*, and eliminating pathogens [55]. In addition, most of the OTCM included in our study have the effect of moving *Qi* and removing food stagnation and are widely used in the treatment of IBS-C, or tonifying *Qi*, invigorating spleen and draining dampness and checking diarrhea for IBS-D according to TCM theories.

Evidence illustrates that some TCM therapies have “two-way adjusting effects” (for example, TCM herbal decoctions, acupuncture, and moxibustion) [57–60], and we briefly divided them into TCM internal therapies (e.g., TCM herbal decoctions and Chinese patent medicine) and TCM external therapies (e.g., acupuncture, moxibustion, acupoint application, and ACE). Modern researches show that ACE plays its role by recovering nerve function, regulating neural reflex, increasing human immunity, improving local circulation, inhibiting the release of inflammatory factors, reducing apoptosis, regulating cellular factor, and improving body metabolism [55]. For instance, evidence shows that ACE could be applied for diarrhea/constipation [24, 61].

Compared with other TCM therapies, ACE could provide patients with lower expense, shorter time of treatment, and longer stimulation sustention with fewer side effects such as local pain and hematoma [62]. As a result, ACE should be widely recognized and accepted for IBS in future. It should be also pointed out that several studies of high quality in recent years approved the efficacy and safety of some TCM external therapies, especially acupoint-based ones, in preventing and controlling some diseases, such as acupuncture for postprandial distress syndrome, acupuncture and acupressure for cancer pain, acupuncture for chronic stable angina, and acupressure combined with TCM footbath for diabetic peripheral neuropathy [63–66]. Such external therapies could reduce the intake of some medications, especially those that may be substantially addictive and with adverse events (e.g., opioid).

In our study, only two of 30 trials reported adverse events related to ACE, and no adverse event was reported on other interventions. The adverse events of ACE were local induration, redness, swelling, and itchiness, which disappeared swiftly after local normal acupuncture and hot compress or local application of iodophor. Results of Wang and colleagues demonstrated that adverse events were found in 70 of 331 patients (21.1%) who seek ACE, including discomfort, body temperature rising after treatment, local hematoma, subcutaneous hemorrhage, swelling, induration, pain, pruritus, redness, and fever [67]. Fainting during the treatment of ACE (1 case) was reported in one trial [68].

As for study quality and risk of bias, all the 30 trials are RCTs, but only one of them implied placebo control. Randomization method was clear and appropriate in eight trials, while it was of unclear risk of bias for the other 22 trials. Allocation concealment and blinding method were of unclear risk of bias in most trials. No study reported drop-out, and a protocol or registration ahead of experiment was reported in only two trials. As a result, more double-blind, prospective, randomized, placebo-controlled trials of ACE as a therapy for IBS with different subtypes are urgently needed.

## 5. Limitations

There are several limitations in our systematic review and meta-analysis. First, most of the trials included were of moderate-to-high risk of bias, with reasons, such as, without mentioning details, random sequence generation method, allocation concealment, and blinding of participants, personnel, and outcome assessment. This is the main reason for low quality of the included trials. Second, interventions and follow-up periods were short among most of the trials, while longer treatment duration and follow-up periods for IBS, a chronic and recurrent disorder, are essential and required. Finally, assessment of publication bias was not applicable in our study, for no more than 10 trials meeting the criteria were included in each comparison.

Generally, more RCTs of high quality and large sample size and with longer treatment duration and follow-up periods are needed to further improve and update our evidence.

## 6. Conclusion

This systematic review and meta-analysis demonstrated that applying ACE alone or plus OWM or plus other ABT or plus OTCM may be more effective for the treatment of IBS-C and IBS-D than OWM alone. ACE plus OTCM or plus other ABT or plus OWM exhibited favorable improvements compared with the control groups (OTCM, other ABT, or OWM alone correspondingly) for IBS-D. Adverse events of ACE were local induration, redness, swelling, and itchiness, but they were all mild and disappeared swiftly with ordinary local intervention. There is an urgent need for RCTs of high quality and large sample size and with longer treatment duration and follow-up periods of ACE for IBS.

## Figures and Tables

**Table 1:** Summary of findings for IBS-C.

Intervention	Outcome	No. of trials	Participants	Effect size (RR/MD)	95% CI	*P* value of effect size	*I* ^2^ value (%)
ACE vs. OWM	Recovery rate	2	198	5.50	1.99 to 15.17	<0.001	0
	Accumulative marked effective rate			3.46	2.03 to 5.90		
	Accumulative effective rate			1.30	1.12 to 1.50		
	Abdominal pain score			−0.53	−0.55 to −0.51		58
	Abdominal distention score			−0.26	−0.29 to −0.23		0

ACE + OAT vs. OWM	Recovery rate	3	196	2.73	1.22 to 6.09	0.01	0
	Accumulative marked effective rate	1	68	1.44	0.82 to 2.51	0.2	NA
	Accumulative effective rate	3	196	1.27	1.08 to 1.49	<0.01	0

ACE + OTCM vs. OWM	Anorectal resting pressure	2	110	−2.81	−5.32 to −0.3	0.03	0
	Rectal maximum tolerance capacity			−19.3	−32.56 to −6.04	<0.01	77

ACE: acupoint catgut embedding; OWM: oral western medicine; OAT: other acupoint therapies; OTCM: oral traditional Chinese medicine; NA: not applicable.

**Table 2:** Summary of findings for IBS-D.

Intervention	Outcome	No. of trials	Participants	Effect size (RR/MD)	95% CI	*P* value of effect size	*I* ^2^ value (%)
ACE vs. OWM	Recovery rate	3	181	2.16	1.33 to 3.53	<0.01	0
	Accumulative marked effective rate	4	241	1.44	1.14 to 1.83	<0.01	8
	Accumulative effective rate			1.27	1.12 to 1.44	<0.01	0
	Recurrence rate (3 months)	2	101	0.49	0.07 to 3.24	0.46	60
	Abdominal pain score	2	121	−0.75	−1.41 to −0.1	0.02	76
	Defecation frequency score			−0.93	−3.52 to 1.65	0.48	99
	Mucinous stool score			0.13	−0.08 to 0.33	0.23	15

ACE + OAT vs. OWM	Accumulative marked effective rate	2	131	1.8	1.23 to 2.62	<0.01	0
	Accumulative effective rate			1.33	1.09 to 1.62	<0.01	35

ACE + OTCM vs. OWM	Recovery rate	3	191	3.36	0.35 to 31.93	0.29	77
	Accumulative marked effective rate			1.87	1.24 to 2.81	<0.01	47
	Accumulative effective rate			1.31	1.15 to 1.5	<0.01	0

ACE + OTCM vs. OTCM	Recovery rate	2	120	1.82	1.14 to 2.92	0.01	0
	Accumulative marked effective rate	2	126	1.31	0.98 to 1.74	0.07	0
	Accumulative effective rate	3	206	1.11	0.99 to 1.24	0.07	0
	Recurrence rate (6 months)	2	120	0.65	0.33 to 1.26	0.2	0

ACE + OAT vs. OAT	Accumulative marked effective rate	2	248	1.32	1.12 to 1.56	<0.01	0
	Accumulative effective rate			1.23	1.11 to 1.35	<0.01	0

ACE + OWM vs. OWM	Recovery rate	2	165	2.72	0.76 to 9.68	0.12	Not applicable
	Accumulative marked effective rate			5.09	0.27 to 94.99	0.28	78
	Accumulative effective rate			1.46	1.21 to 1.77	<0.01	0

ACE: acupoint catgut embedding; OWM: oral western medicine; OAT: other acupoint therapies; OTCM: oral traditional Chinese medicine; NA: not applicable.

## Data Availability

The data are available upon request to the corresponding author.

## Abbreviations

IBS-D: Diarrhea-predominant irritable bowel syndrome
IBS-C: Constipation-predominant irritable bowel syndrome
ACE: Acupoint catgut embedding
OWM: Oral western medicine
ABT: Acupoint-based therapy
OTCM: Oral traditional Chinese medicine
RR: Risk ratio
MD: Mean difference
CI: Confidence interval.

## Supplementary Materials

Supplementary Materials Detailed characteristics of included trials (IBS-C and IBS-D), the flow diagram of the study selection process, and forest plots of the comparisons in our study are listed in supplementary materials document.
