# Supplementary material for: Efficacy and Safety of Acupoint Catgut Embedding for Diarrhea-Predominant Irritable Bowel Syndrome and Constipation-Predominant Irritable Bowel Syndrome: A Systematic Review and Meta-Analysis
Source: Evid Based Complement Alternat Med. 2020 Nov 27;2020:5812320. doi: 10.1155/2020/5812320 (PMC7735853; doi:10.1155/2020/5812320)
Supplement: Supplementary Materials — Detailed characteristics of included trials (IBS-C and IBS-D), the flow diagram of the study selection process, and forest plots of the comparisons in our study are listed in supplementary materials document. [file 5812320.f1.pdf]

table S1: Detailed characteristics of included trials (IBS-C) 5

table S2: Detailed characteristics of included trials (IBS-D) 9

figure S1: The flow diagram of the study selection process 10

figure S2: Acupoint catgut embedding (ACE) alone versus oral Western medicine (OWM) alone-1 for constipation -predominant irritable bowel syndrome (IBS-C) 11

figure S3: Acupoint catgut embedding (ACE) alone versus oral Western medicine (OWM) alone-2 for constipation -predominant irritable bowel syndrome (IBS-C) 12

figure S4: Acupoint catgut embedding (ACE) alone versus oral Western medicine (OWM) alone-1 for diarrhea-predominant irritable bowel syndrome (IBS-D) 13

figure S5: Acupoint catgut embedding (ACE) alone versus oral Western medicine (OWM) alone-2 for diarrhea-predominant irritable bowel syndrome (IBS-D) 14

figure S6: Acupoint catgut embedding (ACE) plus other acupoint-based therapy (ABT) versus oral Western medicine (OWM) alone for constipation -predominant irritable bowel syndrome (IBS-C) 15

figure S7: Acupoint catgut embedding (ACE) plus other acupoint-based therapy (ABT) versus oral Western medicine (OWM) alone for diarrhea-predominant irritable bowel syndrome (IBS-D) 16

figure S8: Acupoint catgut embedding (ACE) plus oral traditional Chinese medicine (OTCM) versus oral Western medicine (OWM) alone for constipation -predominant irritable bowel syndrome (IBS-C) 17

figure S9: Acupoint catgut embedding (ACE) plus oral traditional Chinese medicine (OTCM) versus oral Western medicine (OWM) alone for diarrhea-predominant irritable bowel syndrome (IBS-D)........ 18

figure S10: Acupoint catgut embedding (ACE) plus oral traditional Chinese medicine (OTCM) versus OTCM alone for diarrhea-predominant irritable bowel syndrome (IBS-D) 19

figure S11: Acupoint catgut embedding (ACE) plus other acupoint-based therapy (ABT) versus other ABT alone for diarrhea-predominant irritable bowel syndrome (IBS-D)........ 20

figure S12: Acupoint catgut embedding (ACE) plus oral Western medicine (OWM) versus OWM alone for diarrhea-predominant irritable bowel syndrome (IBS-D)........ 21

Study	Age (E/C)	Duration (E/C)	Male (E/C)	Female (E/C)	Interventions (E/C)	Period of treatment (E/C)	Outcomes	Adverse events (E/C)
Liang 2010a	20-70(mean=53.6)/22-68(mean=51.3)Y	0.5-8/0.5-8Y	10/8	20/17	ACE (qw) + Tai chi chuan/ Mosapride tablets (tid)	2M	1. Recovery rate; 2. Accumulative effective rate.	NR
Liu 2019	A*	B*	9/14	38/33	ACE (1 per 2W)/Calcium polycarbophil tablets (tid)	1M	1. Recovery rate; 2. Accumulative marked effective rate; 3. Accumulative effective rate; 4. Total symptom score; 5. Main symptom score; 6. Defecation frequency score; 7. Defecation difficulty score; 8. Fecal shape score; 9. Defecation time score; 10. Abdominal pain score; 11. Abdominal distention score; 12. Thirst score; 13. Palpitation and fatigue score; 14. Insomnia and dreaminess score; 15. Chest fullness score; 16. Belching and sighing score; 17. Sweating and shortness of breath score; 18. Inappetence score; 19. Fidgety and anxious score; 20. Total efficiency after one month.	N/N
Gao 2016	20-62(33.00±12.20)/22-65(35.00±11.79)Y	6-120(mean=50.23)/22-68(mean=54.77)M	14/12	19/21	ACE (qw) + auricular point sticking (tid)/ Mosapride ctrate capsules (tid)	4W	1. Recovery rate; 2. Accumulative effective rate; 3. Cleveland clinic score (total).	N/N
Chai 2019	(34.23±10.17)/(33.71±9.74)Y	1-10(2.90±0.67)/1-12(3.12±0.59)Y	12/14	23/21	ACE (1 per 15D) + Lactulose oral	60D	1. Recovery rate; 2. Accumulative marked effective rate; 3. Accumulative effective rate; 4. Clinical symptom score; 5. Irritable Bowel Syndrome Quality of Life (IBS-QOL) questionnaire score.	NR

					liquid (tid)/ Lactulose oral liquid (tid)			
Xu 2018	18-74(51.21±1.58)/18-72(49.28±2.29)Y	(6M-11)/(6M-10)Y	12/10	19/21	ACE (qw) + auricular point sticking / Mosapride ctrate capsules (tid)	2W	1. Recovery rate; 2. Accumulative effective rate; 3. Constipation related symptom score.	NR
Zou 2010	19-58(42±3)Y	8(M)-4Y	28	32	ACE (qw) + Shugan Daozhi Decoction (TCM, tid)/Itopride hydrochloride tablets (tid)	4W	1. Abdominal pain score; 2. Abdominal distention score; 3. Stool frequency score; 4. Stool character score; 5. Threshold of anorectum motor and sensory ability; 6. Recurrence rate (1 month).	NR
Hao 2013	16-58(mean=36.5)/21-60(mean=40.1)Y	6(M)-12(mean=3.8)/6(M)-9(mean=4.1)Y	9/8	16/17	ACE (q.15.d) + Zhizhu granules (TCM, bid)/ Mosapride tablets (tid)	1M	1. Recovery rate; 2. Accumulative marked effective rate; 3. Accumulative effective rate.	N/N
Dong 2011	C*	NR	11/10	19/20	ACE (qw) + diet therapy /sham ACE (qw) + diet	6W	1. Somatization symptom score; 2. Obsession symptom score; 3. Interpersonal sensitivity score; 4. Depression score; 5. Anxiety score; 6. Hostility symptom score; 7. Terror symptom score; 8. Paranoia score; 9. Psychosis symptom score; 10. Score of other condition; 11. Clinical	N/N

					therapy		constipation symptom score.	
Jin 2014	18-65(mean=40.3)/20-63(mean=39.6)Y	6(M)-10(mean=3.2)/1-8(mean=3.4)Y	14/15	18/17	ACE (1 per 2W)/Domperidone tablets (tid)	4W	1. Recovery rate; 2. Accumulative marked effective rate; 3. Accumulative effective rate; 4. Abdominal distention score; 5. Abdominal pain score; 6. Defecation frequency score; 7. Defecation difficulty score; 8. Incomplete defecation score; 9. Total symptom score.	NR
Liang 2010b	20-75(mean=52)/22-70(mean=54)Y	(0.5-7)Y	15/10	23/20	ACE (qw) + finger-pressing (qd)/ Mosapride citrate dispersible tablets (tid)	4W/5W	1. Recovery rate; 2. Accumulative marked effective rate; 3. Accumulative effective rate.	NR
Hao 2015	16-58(mean=34.5)/21-60(mean=40.1)Y	3(M)-12(mean=3.2)/6(M)-9(mean=4.1)Y	9/8	16/17	ACE (NR) + Zhizhu Decoction (TCM, bid)/ Mosapride capsule (tid)	*(1M)	1. Anorectal resting pressure; 2. Anorectal pressure during defecation simulation; 3. Rectal response to volume expansion stimulation.	NR
Y: Yeay; M: Month; W: Week; D: Day; NR: Not reported; N: None; ACE: Acupoint catgut embedding; A*: 14 cases (18-40Y), 16 cases (41-60Y), 17 cases (61-85Y) in the experimental group, and 11 cases (18-40Y), 21 cases (41-60Y), 15 cases (61-85Y) in the control group; B*: 15 cases (12-18M), 16 cases (19-36M), 16 cases (>36M) in the experimental group, and 11 cases (12-18M), 21 cases (19-36M), 15 cases (>36M) in the control group; C*: 12 cases (18-35Y), 10 cases (36-55Y), 8 cases (56-65Y) in the experimental group, and 10 cases (18-35Y), 11 cases (36-55Y), 9 cases (56-65Y) in the control group.								

table S1: Detailed characteristics of included trials (IBS-C)

Study	Age (E/C)	Duration (E/C)	Male (E/C)	Female (E/C)	Interventions (E/C)	Period of treatment (E/C)	Outcomes	Adverse events (E/C)
Chai 2018	25-65Y	1-22Y	58	70	ACE (qw)+ Fire needle therapy (1 per 3D)/Normal acupuncture (qd)	NR	1. Recovery rate; 2. Accumulative marked effective rate; 3. Accumulative effective rate.	NR
Deng 2017	21-63(42.56±1.7)Y	4(M)-2(0.85±0.14)Y	33	27	ACE (qw)+ Compound Zhixie Powder (TCM, bid)/ Trimebutine Maleate (tid)	8W	1. Recovery rate; 2. Accumulative marked effective rate; 3. Accumulative effective rate; 4. Total symptom score.	NR
Guo 2011	22-58(mean=38.5)/24-61(mean=39.6)Y	1-10(mean=7.5)/1.5-11(mean=7.9)Y	18/16	22/24	ACE (1 per 15D)+ Tongxie Yaofang Decoction (TCM, bid)/ Tongxie Yaofang Decoction TCM, (bid)	1M	1. Recovery rate; 2. Accumulative effective rate; 3. Recurrence rate (6 month).	NR
Hong 2011	19-65(42.7±9.5)/18-69(45.5±9)Y	0.9-15(5.2±0.85)/1.2-10.6(6.3±1.1)Y	16/13	14/17	ACE (1 pre 10D)/ Pividromide tablets (tid)	30D	1. Recovery rate; 2. Accumulative marked effective rate; 3. Accumulative effective rate; 4. Recurrence rate (3 month).	NR
Huang 2008	20-60(30.21±5.23)/20-62(32.21±4.26)Y	2-48(9.23±2.96)/4-50(8.32±2.51)M	10/12	30/28	ACE (1 pre 10D)/ Otibromide tablets (tid)	30D	1. Recovery rate; 2. Accumulative marked effective rate; 3. Accumulative effective rate; 4. Abdominal pain score; 5. Diarrhea score; 6. Defecation frequency score; 7. Mucinous stool score.	NR

Liu 2013	(37±8.12)/(35±8.66)Y	A*	13/12	17/17	ACE (qw)/ Tongxie Yaofang Decoction (TCM, qd)	6W	1. Recovery rate; 2. Accumulative marked effective rate; 3. Accumulative effective rate; 4. Total symptom score; 5. Diarrhea score; 6. Abdominal pain and distention score; 7. Tinnitus score; 8.Glomus and fullness score; 9. Inappetence score; 10. Languid score; 11. Fatigue score; 12. Thirst score; 13. Belching score; 14. Scanty yellowish urine score; 15. Fear of cold score; 16. Limp aching lumbus and knees score.	Y/N
Lu 2015	20-62(32.21±4.26)Y	1(M)-2(0.81±0.12)Y	31	49	ACE (qw)+ Shenling Baizhu Powdercombined with Tongxie Yaofang Decoction (TCM, bid)/ Trimebutine (tid)	8W	1. Accumulative marked effective rate; 2. Accumulative effective rate (and the two rates of six months and one year after treatment).	NR
Su 2008	18-70Y	6(M)-20Y	46	40	ACE (1 per 15D)+ Anchang Decoction (TCM, qd)/ Anchang Decoction (TCM, qd)	2-3M	1. Accumulative marked effective rate; 2. Accumulative effective rate;	NR
Su 2019	19-65(43.3±9.2)/18-65(44±9.5)Y	0.5-10(3.6±1.1)/0.4-8(3.1±0.9)M	17/15	23/25	ACE (NR)+ Moxibustion at the Governor vessel (qw)/ Pividromide tablets (tid)	4W	1. Recovery rate; 2. Accumulative marked effective rate; 3. Accumulative effective rate; 4. Abdominal pain score; 5. Abdominal distention score; 6. Score of incomplete defecation; 7. Defecation frequency score; 8. Stool consistency score; 9. IBS-QOL score.	N/N
Wang	(37.93±11.45)/(39.1±11.8)Y	(5.23±7.35)/(4.33±3.93)Y	25/25	35/35	ACE (qw)+ acupoint	4W	1. Accumulative marked effective rate; 2.	NR

2017					application (qod)/ acupoint application (qod)		Accumulative effective rate; 3. Abdominal distention score; 4. Abdominal pain score; 5. Diarrhea score.	
Yao 2012	B*	NR	17/20	13/10	ACE (qw)+ Trimebutine Maleate (tid)/ Trimebutine Maleate (tid)	2W	1. Recovery rate; 2. Accumulative marked effective rate; 3. Accumulative effective rate; 4. Total symptom score.	N/N
Ying 2009	mean=45Y	1(M)-6Y	36	15	ACE (NR)+ Aconite cake-partitioned moxibustion (NR)/ Montmorillonite powder (tid)+ Oryzanol (tid)+ Trimebutine Maleate (tid)	NR	1. Accumulative marked effective rate; 2. Accumulative effective rate;	NR
Zeng 2013 a	NR	NR	C*		ACE (qw)+ Buzhong Yiqi Decoction (bid)/ Buzhong Yiqi Decoction (bid)	6W	1. Recovery rate; 2. Accumulative marked effective rate; 3. Accumulative effective rate; 4. Abdominal pain score; 5. Abdominal distention score; 6. Defecation frequency score; 7. Mucinous stool score; 8. Recurrence rate (1 month); 9. Recurrence rate (3 month); 10. Recurrence rate (6 month).	NR
Zeng 2013 b			D*		ACE (qw)+ Buzhong Yiqi Decoction (bid)/ Montmorillonite powder (tid)			
Zeng 2013 c			E*		ACE (qw)/ Buzhong Yiqi Decoction (bid)			
Zeng 2013 d			F*		ACE (qw)/ Montmorillonite powder			

					(tid)			
Zeng 2013 e			G*		ACE (qw)/ ACE (qw)+ Buzhong Yiqi Decoction (bid)			
Zhao 2014	(41.43±11.24)/(40.77±11.13) Y	(14.43±5.67)/(14.8±6.49) M	12/13	18/17	ACE (1 per 3W)/ Peifeikang capsule (tid)+ Otibromide tablets (tid)	6W	1. Accumulative marked effective rate; 2. Accumulative effective rate; 3. Stool classification; 4. Total symptom score.	N/N
Zhao 2019	22-60(41.98±10.72)/20-55(39.42±9.38)Y	1-9.5(3.82±1.84)/1.5-10(3.79±1.84)Y	13/15	39/38	ACE (1 per 2W)+ Trimebutine Maleate (tid)+ Bifidobacterium triple viable enteric coated capsule (tid)/ Trimebutine Maleate (tid)+ Bifidobacterium triple viable enteric coated capsule (tid)	8W	1. Recovery rate; 2. Accumulative marked effective rate; 3. Accumulative effective rate; 4. Total symptom score; 5. Abdominal pain score; 6. Abdominal distention score; 7. Stool consistency score; 8. Total score of symptom severity; 9. Recurrence rate (3 month).	Y/N
Y: Yeay; M: Month; W: Week; D: Day; NR: Not reported; N: None; ACE: Acupoint catgut embedding; A*: 4 cases (<0.5 year), 14 cases (0.5-3 years), 12 cases (>3 years) in the experimental group and 3 cases (<0.5 year), 16 cases (0.5-3 years) 10 cases (>3 years) in the control group; B*: 11 cases (18-29 years old), 9 cases (30-39 years old), 6 cases (40-49 years old), 4 cases (50-65 years old) in the experimental group and 11 cases (18-29 years old), 10 cases (30-39 years old), 5 cases (40-49 years old), 4 cases (50-65 years old) in the control group; C*: 20 cases in the experimental group and 20 cases in the control group; D*: 20 cases in the experimental group and 21 cases in the control group; E*: 20 cases in the experimental group and 20 cases in the control group; F*: 20 cases in the experimental group and 21 cases in the control group; G*:20 cases in the experimental group and 20 cases in the control group.								

table S2: Detailed characteristics of included trials (IBS-D)

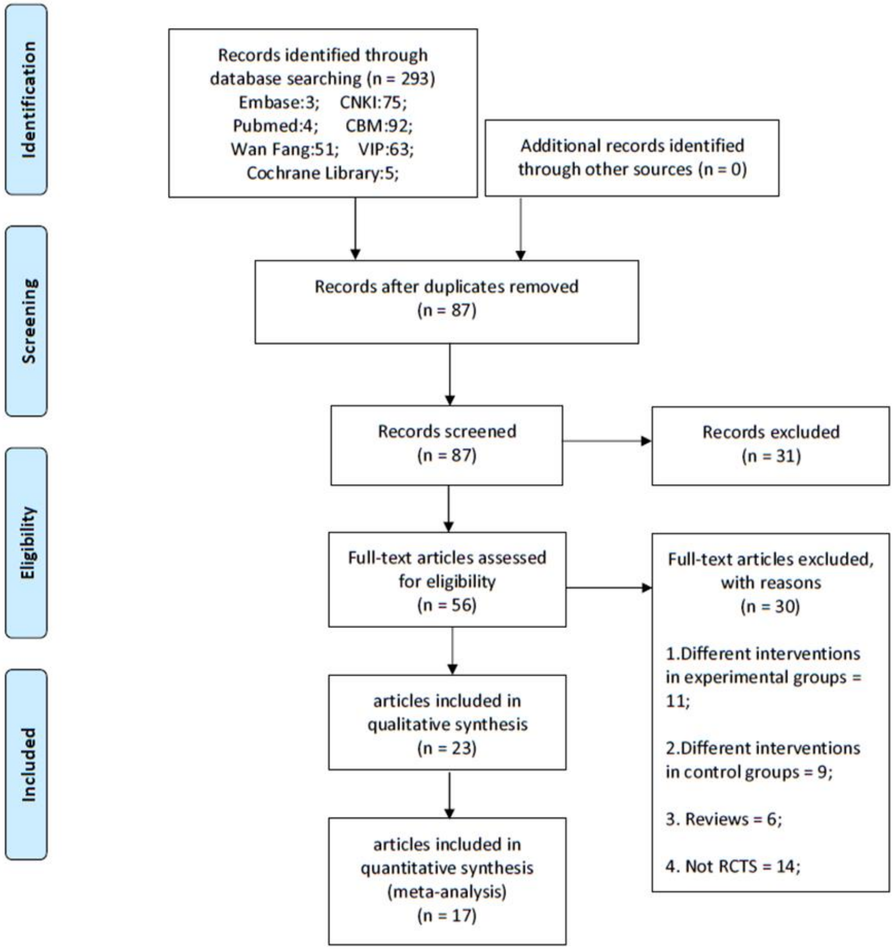

figure S1: The flow diagram of the study selection process

Study or Subgroup	Experimental Events	Experimental Total	Control Events	Control Total	Weight	Risk Ratio M-H, Fixed, 95% CI
**1.1.1 Recovery rate**						
Jin 2014	6	32	1	32	25.0%	6.00 [0.77, 47.05]
Liu 2019	16	47	3	47	75.0%	5.33 [1.66, 17.10]
**Subtotal (95% CI)**		**79**		**79**	**100.0%**	**5.50 [1.99, 15.17]**
Total events	22		4			
Heterogeneity: Chi² = 0.01, df = 1 (P = 0.92); I² = 0%						
Test for overall effect: Z = 3.29 (P = 0.0010)						
**1.1.2 Accumulative marked effective rate**						
Jin 2014	17	32	6	32	46.2%	2.83 [1.28, 6.25]
Liu 2019	28	47	7	47	53.8%	4.00 [1.94, 8.24]
**Subtotal (95% CI)**		**79**		**79**	**100.0%**	**3.46 [2.03, 5.90]**
Total events	45		13			
Heterogeneity: Chi² = 0.40, df = 1 (P = 0.53); I² = 0%						
Test for overall effect: Z = 4.56 (P < 0.00001)						
**1.1.3 Accumulative effective rate**						
Jin 2014	28	32	21	32	36.8%	1.33 [1.00, 1.77]
Liu 2019	46	47	36	47	63.2%	1.28 [1.08, 1.50]
**Subtotal (95% CI)**		**79**		**79**	**100.0%**	**1.30 [1.12, 1.50]**
Total events	74		57			
Heterogeneity: Chi² = 0.07, df = 1 (P = 0.79); I² = 0%						
Test for overall effect: Z = 3.48 (P = 0.0005)						

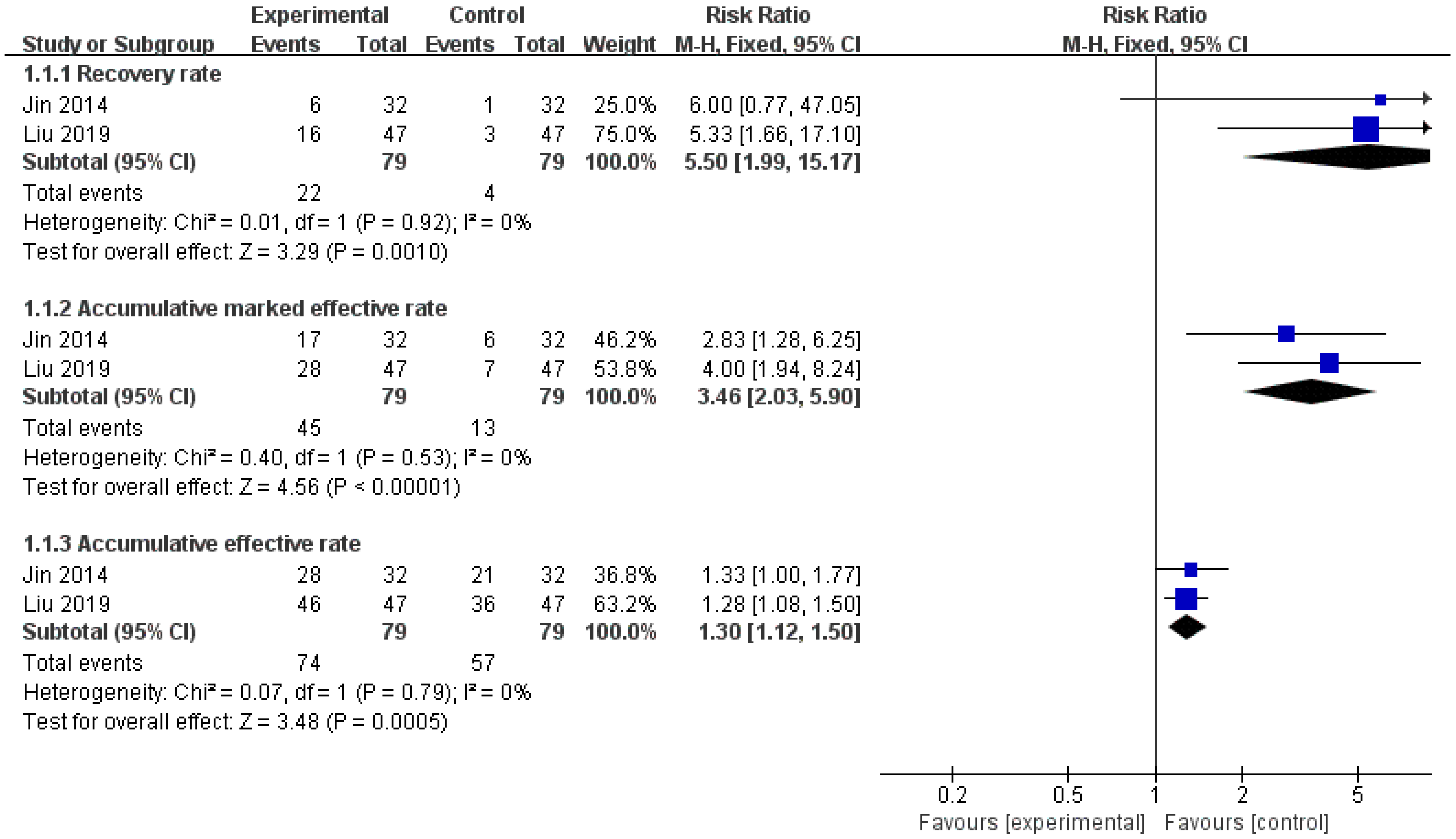

figure S2: Acupoint catgut embedding (ACE) alone versus oral Western medicine (OWM) alone-1 for constipation -predominant irritable bowel syndrome (IBS-C)

	Experimental			Control				Mean Difference
**Study or Subgroup**	**Mean**	**SD**	**Total**	**Mean**	**SD**	**Total**	**Weight**	**IV, Random, 95% CI**
**1.2.1 Abdominal pain score**								
Jin 2014	0.28	0.46	32	0.59	0.67	32	29.2%	-0.31 [-0.59, -0.03]
Liu 2019	0.0851	0.04114	47	0.617	0.07167	47	70.8%	-0.53 [-0.56, -0.51]
**Subtotal (95% CI)**			**79**			**79**	**100.0%**	**-0.47 [-0.66, -0.27]**
Heterogeneity: Tau² = 0.01; Chi² = 2.37, df = 1 (P = 0.12); I² = 58%								
Test for overall effect: Z = 4.63 (P < 0.00001)								
**1.2.2 Abdominal distention score**								
Jin 2014	0.44	0.67	32	0.81	0.78	32	0.8%	-0.37 [-0.73, -0.01]
Liu 2019	0.4255	0.0729	47	0.6809	0.08108	47	99.2%	-0.26 [-0.29, -0.22]
**Subtotal (95% CI)**			**79**			**79**	**100.0%**	**-0.26 [-0.29, -0.23]**
Heterogeneity: Tau² = 0.00; Chi² = 0.39, df = 1 (P = 0.53); I² = 0%								
Test for overall effect: Z = 16.17 (P < 0.00001)								

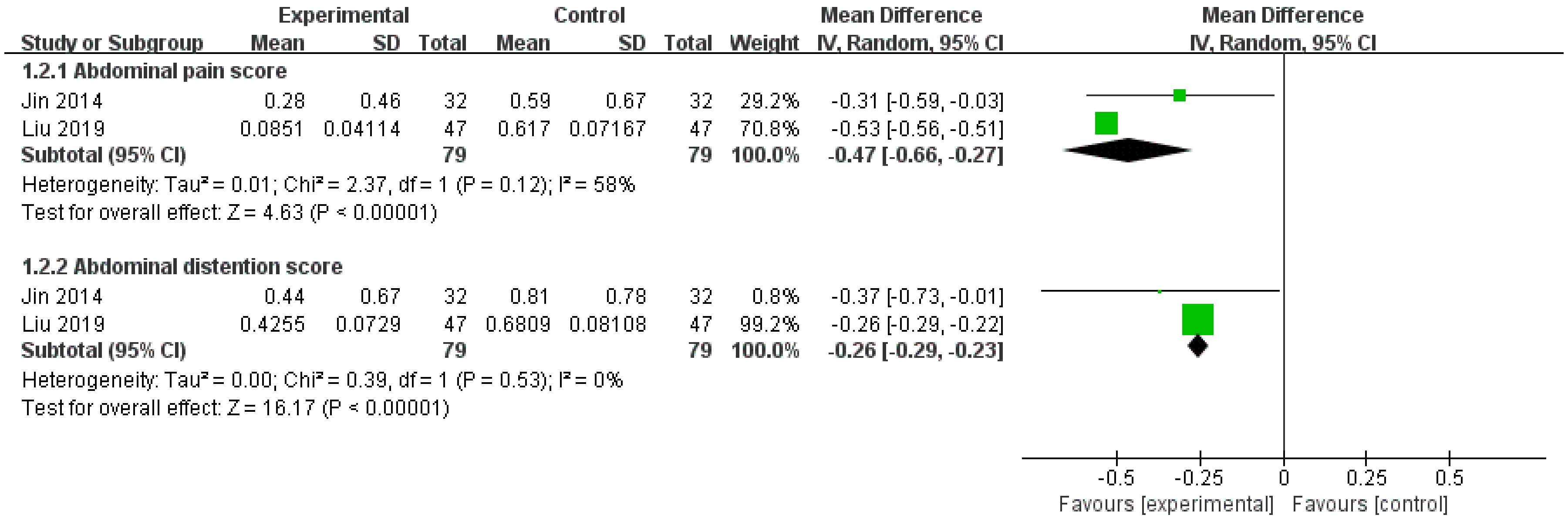

figure S3: Acupoint catgut embedding (ACE) alone versus oral Western medicine (OWM) alone-2 for constipation -predominant irritable bowel syndrome (IBS-C)

Study or Subgroup	Experimental Events	Experimental Total	Control Events	Control Total	Weight	Risk Ratio M-H, Random, 95% CI
**4.1.1 Recovery rate**						
Hong 2011	6	30	2	30	10.4%	3.00 [0.66, 13.69]
Huang 2008	25	40	12	40	85.1%	2.08 [1.23, 3.54]
Zeng 2013 d	2	20	1	21	4.5%	2.10 [0.21, 21.39]
**Subtotal (95% CI)**		**90**		**91**	**100.0%**	**2.16 [1.33, 3.53]**
Total events	33		15			
Heterogeneity: Tau² = 0.00; Chi² = 0.20, df = 2 (P = 0.90); I² = 0%						
Test for overall effect: Z = 3.09 (P = 0.002)						
**4.1.2 Accumulative marked effective rate**						
Hong 2011	19	30	11	30	17.6%	1.73 [1.00, 2.97]
Huang 2008	34	40	26	40	60.7%	1.31 [1.01, 1.70]
Zeng 2013 d	5	20	6	21	5.3%	0.88 [0.32, 2.42]
Zhao 2014	20	30	10	30	16.3%	2.00 [1.14, 3.52]
**Subtotal (95% CI)**		**120**		**121**	**100.0%**	**1.44 [1.14, 1.83]**
Total events	78		53			
Heterogeneity: Tau² = 0.01; Chi² = 3.27, df = 3 (P = 0.35); I² = 8%						
Test for overall effect: Z = 3.02 (P = 0.003)						
**4.1.3 Accumulative effective rate**						
Hong 2011	26	30	17	30	13.3%	1.53 [1.09, 2.16]
Huang 2008	38	40	32	40	54.0%	1.19 [1.00, 1.41]
Zeng 2013 d	16	20	14	21	11.2%	1.20 [0.83, 1.74]
Zhao 2014	28	30	20	30	21.4%	1.40 [1.07, 1.83]
**Subtotal (95% CI)**		**120**		**121**	**100.0%**	**1.27 [1.12, 1.44]**
Total events	108		83			
Heterogeneity: Tau² = 0.00; Chi² = 2.51, df = 3 (P = 0.47); I² = 0%						
Test for overall effect: Z = 3.79 (P = 0.0002)						
**4.1.4 Recurrence rate (3 months)**						
Hong 2011	4	26	11	17	63.9%	0.24 [0.09, 0.63]
Zeng 2013 d	2	16	1	14	36.1%	1.75 [0.18, 17.29]
**Subtotal (95% CI)**		**42**		**31**	**100.0%**	**0.49 [0.07, 3.24]**
Total events	6		12			
Heterogeneity: Tau² = 1.22; Chi² = 2.51, df = 1 (P = 0.11); I² = 60%						
Test for overall effect: Z = 0.74 (P = 0.46)						

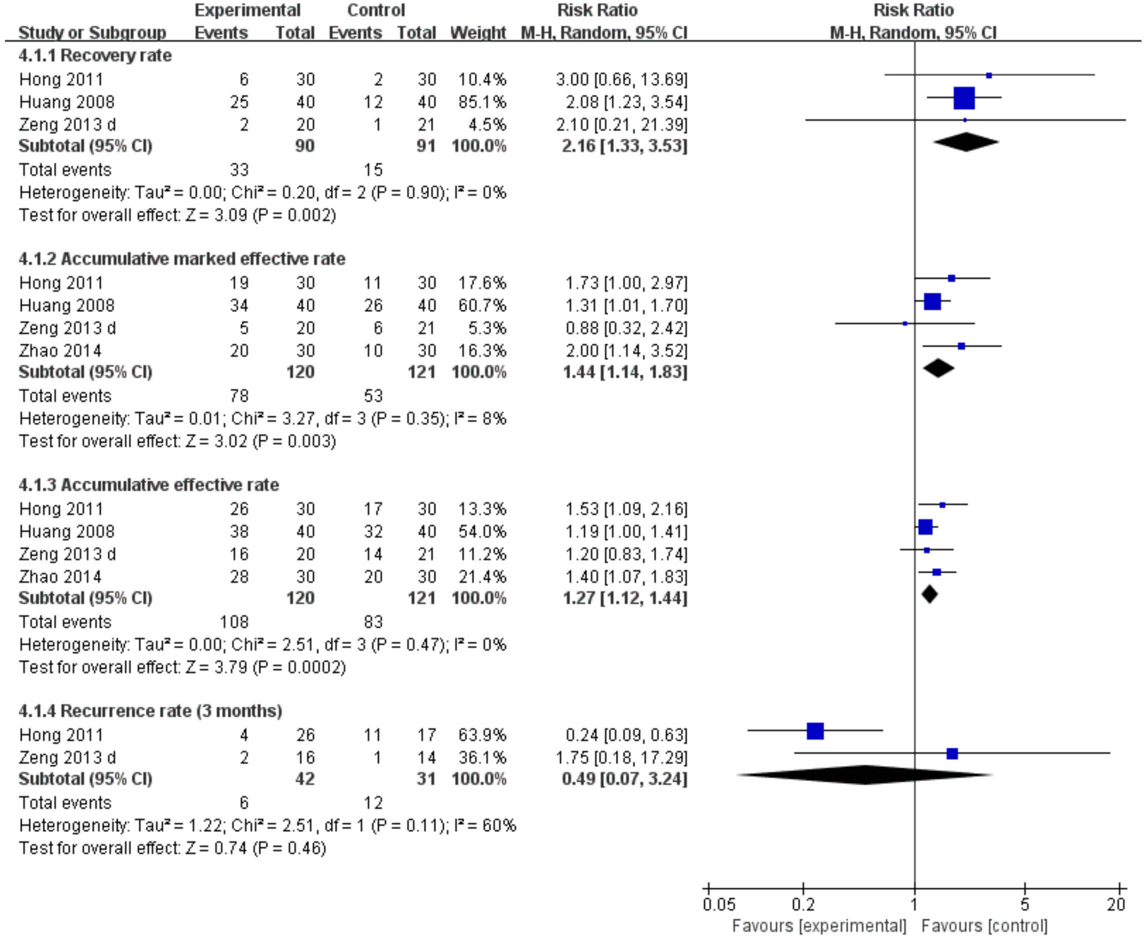

figure S4: Acupoint catgut embedding (ACE) alone versus oral Western medicine (OWM) alone-1 for diarrhea-predominant irritable bowel syndrome (IBS-D)

	Experimental			Control				Mean Difference
Study or Subgroup	Mean	SD	Total	Mean	SD	Total	Weight	IV, Random, 95% CI
**4.2.1 Abdominal pain score**								
Huang 2008	0.85	0.12	40	1.89	0.84	40	57.9%	-1.04 [-1.30, -0.78]
Zeng 2013 d	1.21	0.91	20	1.57	1.02	21	42.1%	-0.36 [-0.95, 0.23]
**Subtotal (95% CI)**			**60**			**61**	**100.0%**	**-0.75 [-1.41, -0.10]**
Heterogeneity: Tau² = 0.18; Chi² = 4.24, df = 1 (P = 0.04); I² = 76%								
Test for overall effect: Z = 2.24 (P = 0.02)								
**4.2.2 Defecation frequency score**								
Huang 2008	0.9	0.29	40	3.15	0.58	40	50.2%	-2.25 [-2.45, -2.05]
Zeng 2013 d	0.85	0.79	20	0.46	0.27	21	49.8%	0.39 [0.03, 0.75]
**Subtotal (95% CI)**			**60**			**61**	**100.0%**	**-0.93 [-3.52, 1.65]**
Heterogeneity: Tau² = 3.46; Chi² = 154.23, df = 1 (P < 0.00001); I² = 99%								
Test for overall effect: Z = 0.71 (P = 0.48)								
**4.2.3 Mucinous stool score**								
Huang 2008	1.66	0.35	40	1.6	0.57	40	71.1%	0.06 [-0.15, 0.27]
Zeng 2013 d	0.83	0.74	20	0.54	0.36	21	28.9%	0.29 [-0.07, 0.65]
**Subtotal (95% CI)**			**60**			**61**	**100.0%**	**0.13 [-0.08, 0.33]**
Heterogeneity: Tau² = 0.00; Chi² = 1.18, df = 1 (P = 0.28); I² = 15%								
Test for overall effect: Z = 1.21 (P = 0.23)								

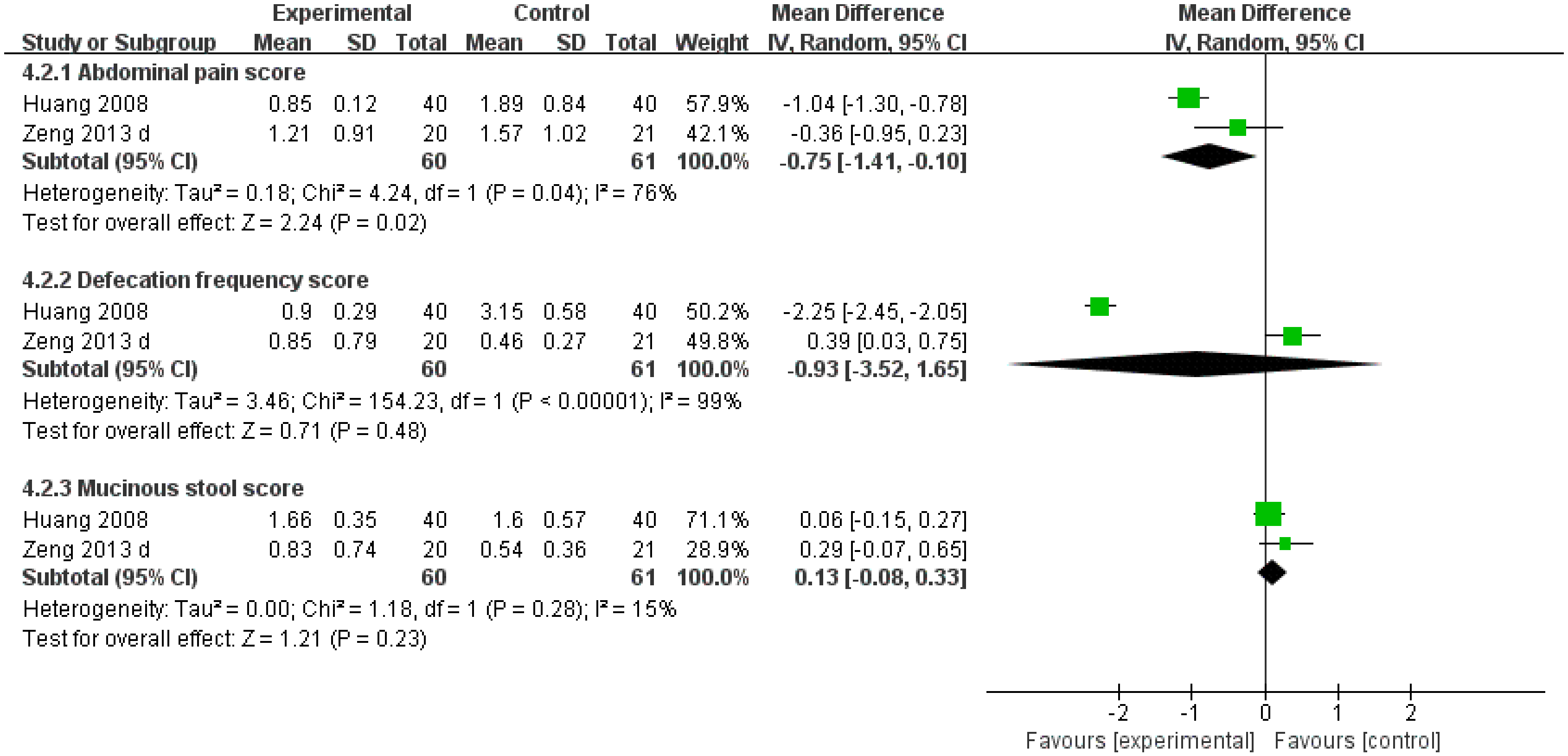

figure S5: Acupoint catgut embedding (ACE) alone versus oral Western medicine (OWM) alone-2 for diarrhea-predominant irritable bowel syndrome (IBS-D)

Study or Subgroup	Experimental Events	Experimental Total	Control Events	Control Total	Weight	Risk Ratio M-H, Fixed, 95% CI
**2.1.1 Recovery rate**						
Gao 2016	7	33	3	33	40.8%	2.33 [0.66, 8.25]
Liang 2010 b	8	38	3	30	45.6%	2.11 [0.61, 7.26]
Xu 2018	6	31	1	31	13.6%	6.00 [0.77, 46.96]
**Subtotal (95% CI)**		**102**		**94**	**100.0%**	**2.73 [1.22, 6.09]**
Total events	21		7			
Heterogeneity: Chi² = 0.79, df = 2 (P = 0.67); I² = 0%						
Test for overall effect: Z = 2.45 (P = 0.01)						
**2.1.2 Accumulative marked effective rate**						
Liang 2010 b	20	38	11	30	100.0%	1.44 [0.82, 2.51]
**Subtotal (95% CI)**		**38**		**30**	**100.0%**	**1.44 [0.82, 2.51]**
Total events	20		11			
Heterogeneity: Not applicable						
Test for overall effect: Z = 1.27 (P = 0.20)						
**2.1.3 Accumulative effective rate**						
Gao 2016	23	33	19	33	29.0%	1.21 [0.84, 1.75]
Liang 2010 b	35	38	21	30	35.8%	1.32 [1.02, 1.69]
Xu 2018	29	31	23	31	35.1%	1.26 [1.00, 1.58]
**Subtotal (95% CI)**		**102**		**94**	**100.0%**	**1.27 [1.08, 1.49]**
Total events	87		63			
Heterogeneity: Chi² = 0.15, df = 2 (P = 0.93); I² = 0%						
Test for overall effect: Z = 2.88 (P = 0.004)						

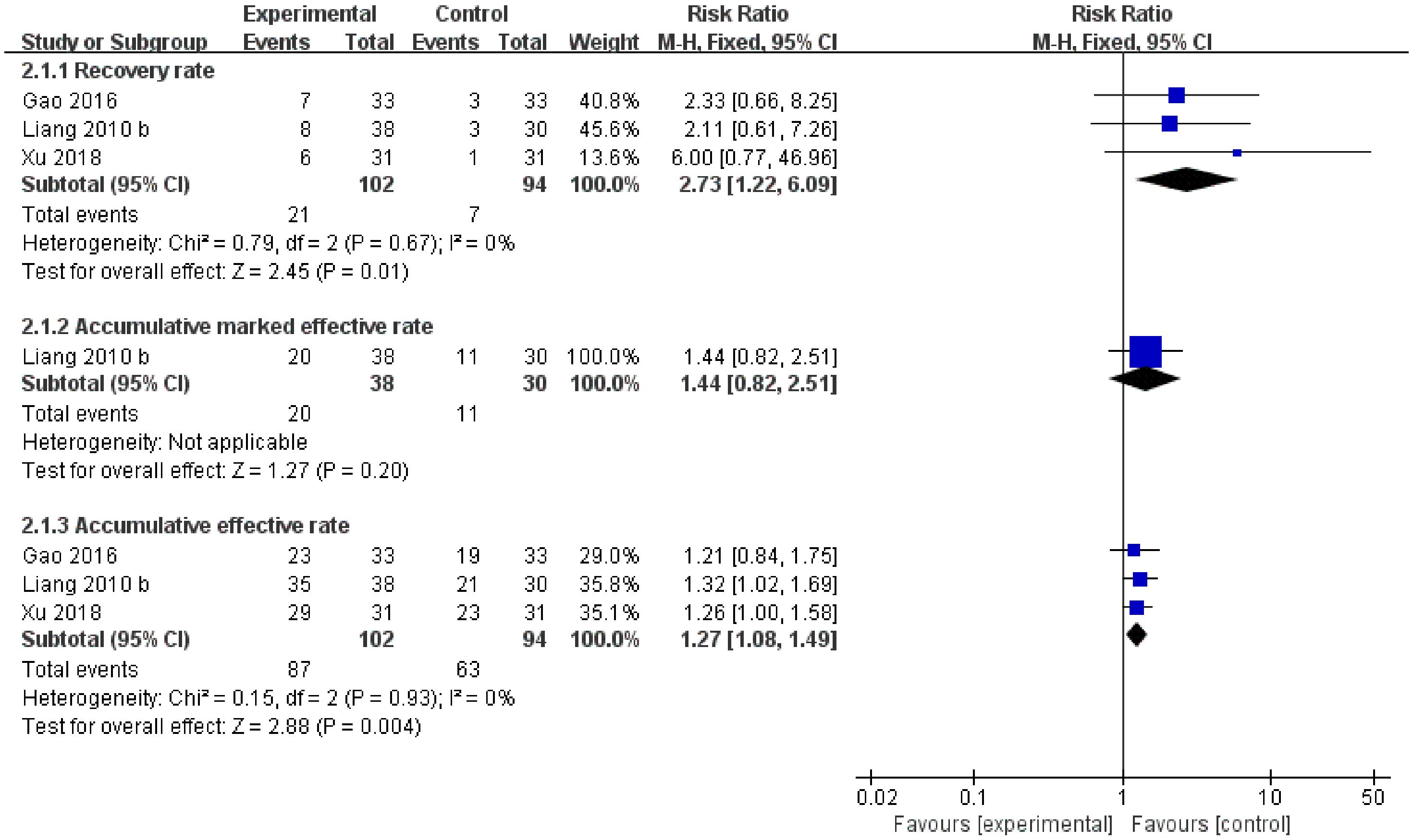

figure S6: Acupoint catgut embedding (ACE) plus other acupoint-based therapy (ABT) versus oral Western medicine (OWM) alone for constipation -predominant irritable bowel syndrome (IBS-C)

Study or Subgroup	Experimental Events	Experimental Total	Control Events	Control Total	Weight	Risk Ratio M-H, Fixed, 95% CI
**5.1.1 Accumulative marked effective rate**						
Su 2019	24	40	15	40	64.8%	1.60 [1.00, 2.57]
Ying 2009	18	26	8	25	35.2%	2.16 [1.16, 4.05]
**Subtotal (95% CI)**		**66**		**65**	**100.0%**	**1.80 [1.23, 2.62]**
Total events	42		23			
Heterogeneity: Chi² = 0.57, df = 1 (P = 0.45); I² = 0%						
Test for overall effect: Z = 3.05 (P = 0.002)						
**5.1.2 Accumulative effective rate**						
Su 2019	35	40	29	40	67.0%	1.21 [0.96, 1.51]
Ying 2009	23	26	14	25	33.0%	1.58 [1.09, 2.30]
**Subtotal (95% CI)**		**66**		**65**	**100.0%**	**1.33 [1.09, 1.62]**
Total events	58		43			
Heterogeneity: Chi² = 1.53, df = 1 (P = 0.22); I² = 35%						
Test for overall effect: Z = 2.85 (P = 0.004)						

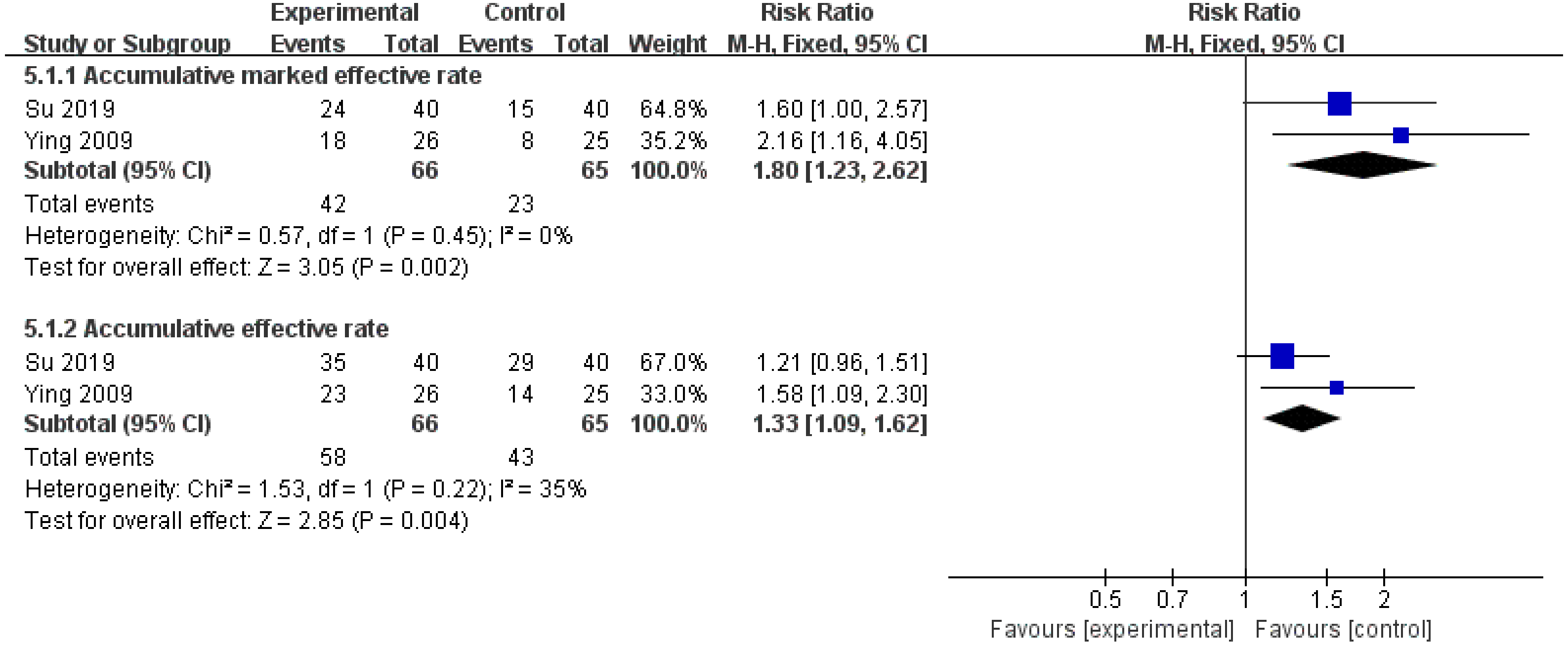

figure S7: Acupoint catgut embedding (ACE) plus other acupoint-based therapy (ABT) versus oral Western medicine (OWM) alone for diarrhea-predominant irritable bowel syndrome (IBS-D)

Study or Subgroup	Experimental Mean	Experimental SD	Experimental Total	Control Mean	Control SD	Control Total	Weight	Mean Difference IV, Random, 95% CI
**3.1.1 Anorectal resting pressure**								
Hao 2015	69.18	16.32	25	75.35	10.38	25	10.9%	-6.17 [-13.75, 1.41]
Zou 2010	59.3	5.2	30	61.7	5.3	30	89.1%	-2.40 [-5.06, 0.26]
**Subtotal (95% CI)**			**55**			**55**	**100.0%**	**-2.81 [-5.32, -0.30]**
Heterogeneity: Tau² = 0.00; Chi² = 0.85, df = 1 (P = 0.36); I² = 0%								
Test for overall effect: Z = 2.20 (P = 0.03)								
**3.1.2 Rectal maximum tolerance capacity**								
Hao 2015	160.13	25.67	25	171.54	15.73	25	42.4%	-11.41 [-23.21, 0.39]
Zou 2010	131.7	11	30	156.8	10.5	30	57.6%	-25.10 [-30.54, -19.66]
**Subtotal (95% CI)**			**55**			**55**	**100.0%**	**-19.30 [-32.56, -6.04]**
Heterogeneity: Tau² = 71.73; Chi² = 4.26, df = 1 (P = 0.04); I² = 77%								
Test for overall effect: Z = 2.85 (P = 0.004)								

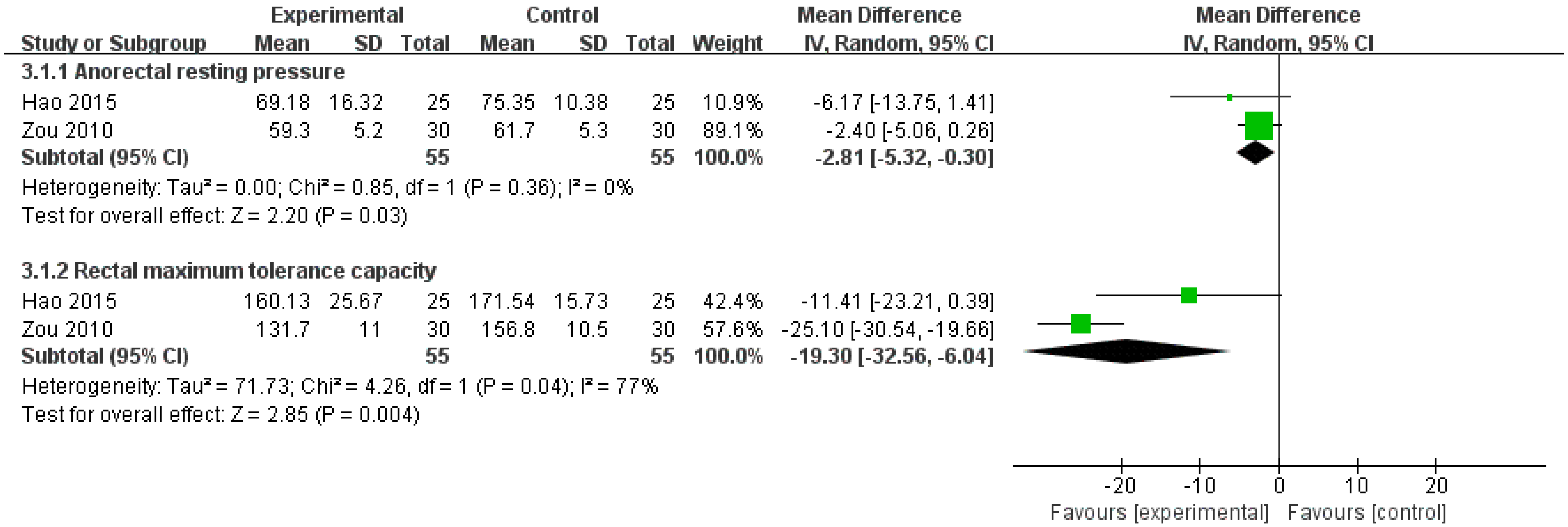

figure S8: Acupoint catgut embedding (ACE) plus oral traditional Chinese medicine (OTCM) versus oral Western medicine (OWM) alone for constipation -predominant irritable bowel syndrome (IBS-C)

Study or Subgroup	Experimental Events	Experimental Total	Control Events	Control Total	Weight	Risk Ratio M-H, Random, 95% CI
**6.1.1 Recovery rate**						
Deng 2017	8	30	6	30	57.1%	1.33 [0.53, 3.38]
Zeng 2013 b	11	20	1	21	42.9%	11.55 [1.64, 81.46]
**Subtotal (95% CI)**		**50**		**51**	**100.0%**	**3.36 [0.35, 31.93]**
Total events	19		7			
Heterogeneity: Tau² = 2.08; Chi² = 4.42, df = 1 (P = 0.04); I² = 77%						
Test for overall effect: Z = 1.06 (P = 0.29)						
**6.1.2 Accumulative marked effective rate**						
Deng 2017	18	30	14	30	35.7%	1.29 [0.79, 2.08]
Lu 2015	33	40	16	40	41.7%	2.06 [1.37, 3.09]
Zeng 2013 b	16	20	6	21	22.6%	2.80 [1.38, 5.70]
**Subtotal (95% CI)**		**90**		**91**	**100.0%**	**1.87 [1.24, 2.81]**
Total events	67		36			
Heterogeneity: Tau² = 0.06; Chi² = 3.80, df = 2 (P = 0.15); I² = 47%						
Test for overall effect: Z = 2.99 (P = 0.003)						
**6.1.3 Accumulative effective rate**						
Deng 2017	27	30	20	30	23.0%	1.35 [1.02, 1.79]
Lu 2015	40	40	31	40	61.1%	1.29 [1.08, 1.53]
Zeng 2013 b	18	20	14	21	15.9%	1.35 [0.96, 1.89]
**Subtotal (95% CI)**		**90**		**91**	**100.0%**	**1.31 [1.15, 1.50]**
Total events	85		65			
Heterogeneity: Tau² = 0.00; Chi² = 0.13, df = 2 (P = 0.94); I² = 0%						
Test for overall effect: Z = 3.95 (P < 0.0001)						

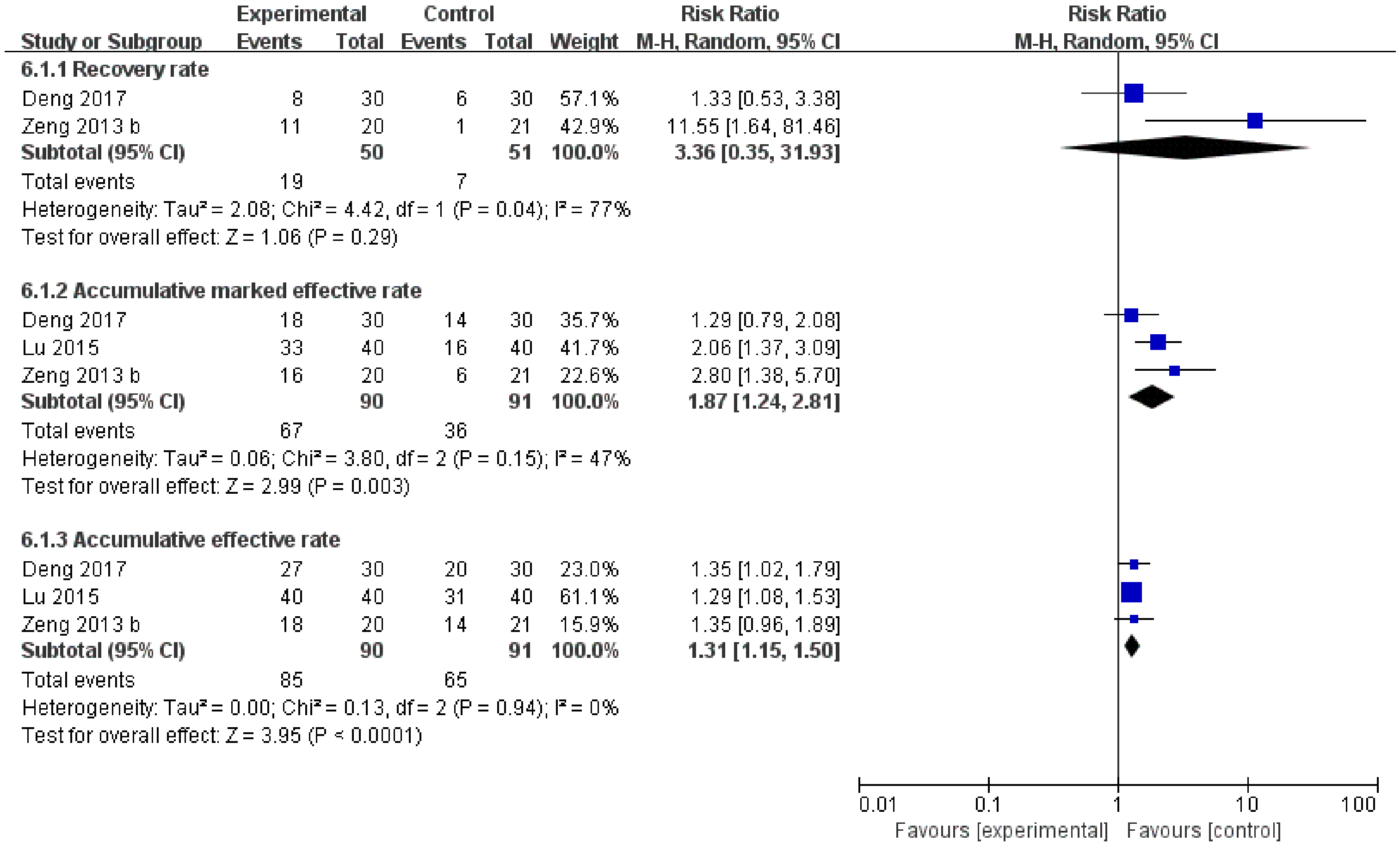

figure S9: Acupoint catgut embedding (ACE) plus oral traditional Chinese medicine (OTCM) versus oral Western medicine (OWM) alone for diarrhea-predominant irritable bowel syndrome (IBS-D)

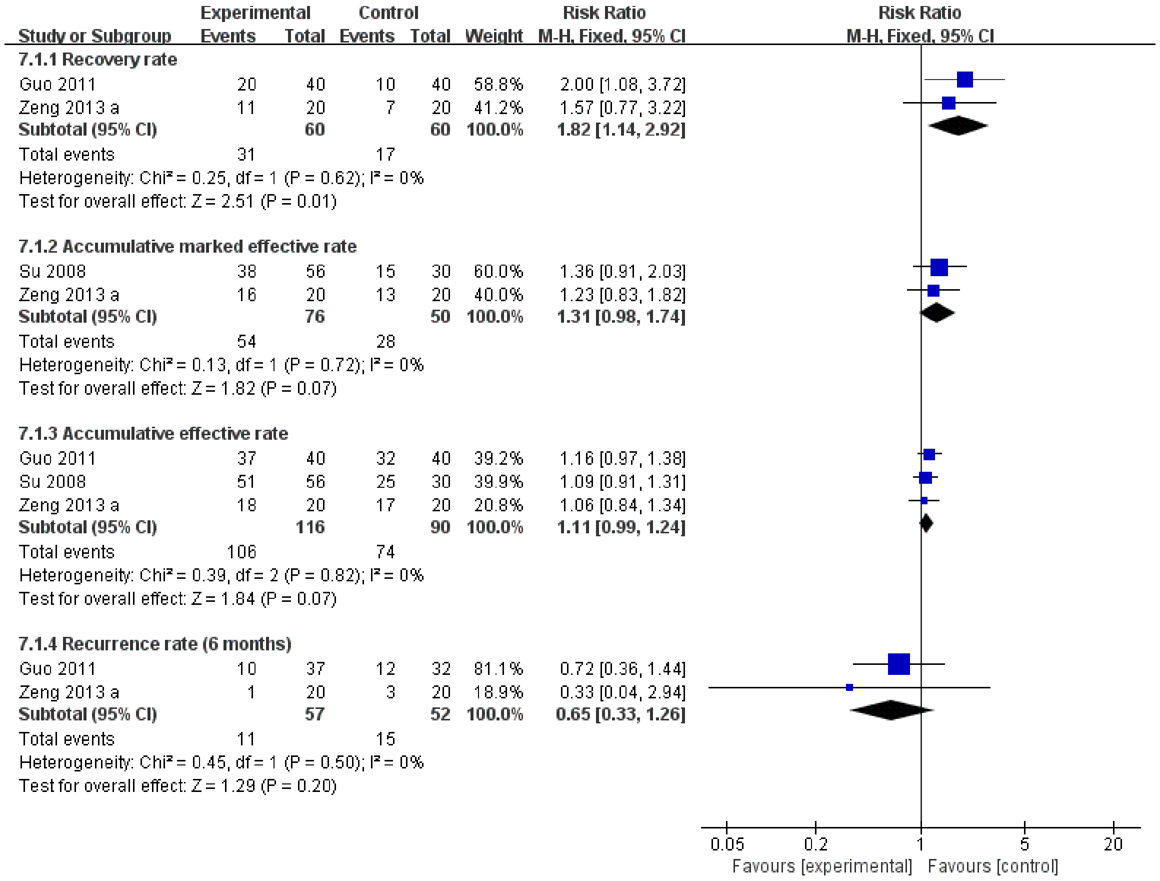

figure S10: Acupoint catgut embedding (ACE) plus oral traditional Chinese medicine (OTCM) versus OTCM alone for diarrhea-predominant irritable bowel syndrome (IBS-D)

Study or Subgroup	Experimental Events	Experimental Total	Control Events	Control Total	Weight	Risk Ratio M-H, Fixed, 95% CI
**8.1.1 Accumulative marked effective rate**						
Chai 2018	60	64	48	64	66.7%	1.25 [1.07, 1.46]
Wang 2017	35	60	24	60	33.3%	1.46 [1.00, 2.13]
**Subtotal (95% CI)**		**124**		**124**	**100.0%**	**1.32 [1.12, 1.56]**
Total events	95		72			
Heterogeneity: Chi² = 0.74, df = 1 (P = 0.39); I² = 0%						
Test for overall effect: Z = 3.28 (P = 0.001)						
**8.1.2 Accumulative effective rate**						
Chai 2018	63	64	53	64	54.6%	1.19 [1.06, 1.33]
Wang 2017	56	60	44	60	45.4%	1.27 [1.08, 1.50]
**Subtotal (95% CI)**		**124**		**124**	**100.0%**	**1.23 [1.11, 1.35]**
Total events	119		97			
Heterogeneity: Chi² = 0.47, df = 1 (P = 0.49); I² = 0%						
Test for overall effect: Z = 4.04 (P < 0.0001)						

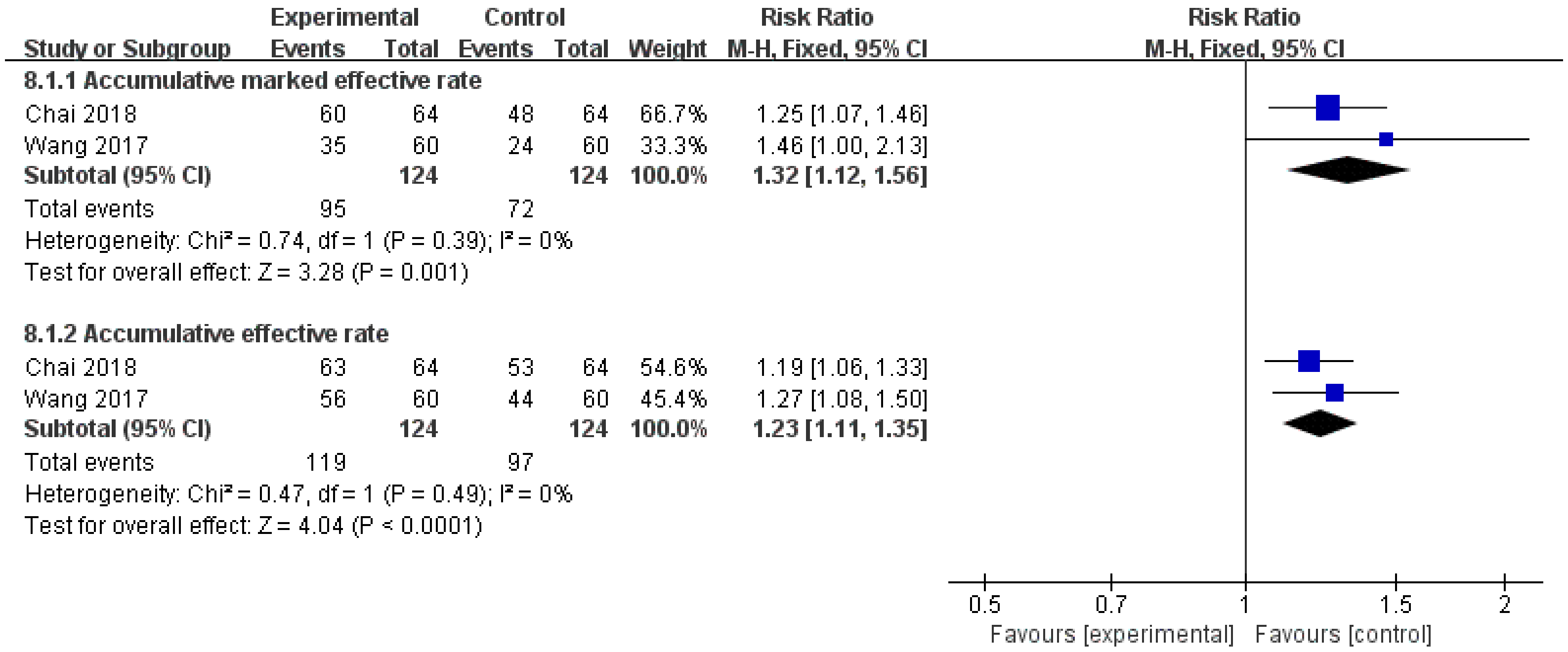

figure S11: Acupoint catgut embedding (ACE) plus other acupoint-based therapy (ABT) versus other ABT alone for diarrhea-predominant irritable bowel syndrome (IBS-D)

Study or Subgroup	Experimental Events	Experimental Total	Control Events	Control Total	Weight	Risk Ratio M-H, Random, 95% CI
**9.1.1 Recovery rate**						
Yao 2012	0	30	0	30		Not estimable
Zhao 2019	8	52	3	53	100.0%	2.72 [0.76, 9.68]
**Subtotal (95% CI)**		**82**		**83**	**100.0%**	**2.72 [0.76, 9.68]**
Total events	8		3			
Heterogeneity: Not applicable						
Test for overall effect: Z = 1.54 (P = 0.12)						
**9.1.2 Accumulative marked effective rate**						
Yao 2012	12	30	0	30	39.5%	25.00 [1.55, 403.99]
Zhao 2019	30	52	17	53	60.5%	1.80 [1.14, 2.84]
**Subtotal (95% CI)**		**82**		**83**	**100.0%**	**5.09 [0.27, 94.99]**
Total events	42		17			
Heterogeneity: Tau² = 3.63; Chi² = 4.51, df = 1 (P = 0.03); I² = 78%						
Test for overall effect: Z = 1.09 (P = 0.28)						
**9.1.3 Accumulative effective rate**						
Yao 2012	27	30	16	30	28.4%	1.69 [1.18, 2.41]
Zhao 2019	46	52	34	53	71.6%	1.38 [1.10, 1.73]
**Subtotal (95% CI)**		**82**		**83**	**100.0%**	**1.46 [1.21, 1.77]**
Total events	73		50			
Heterogeneity: Tau² = 0.00; Chi² = 0.90, df = 1 (P = 0.34); I² = 0%						
Test for overall effect: Z = 3.92 (P < 0.0001)						

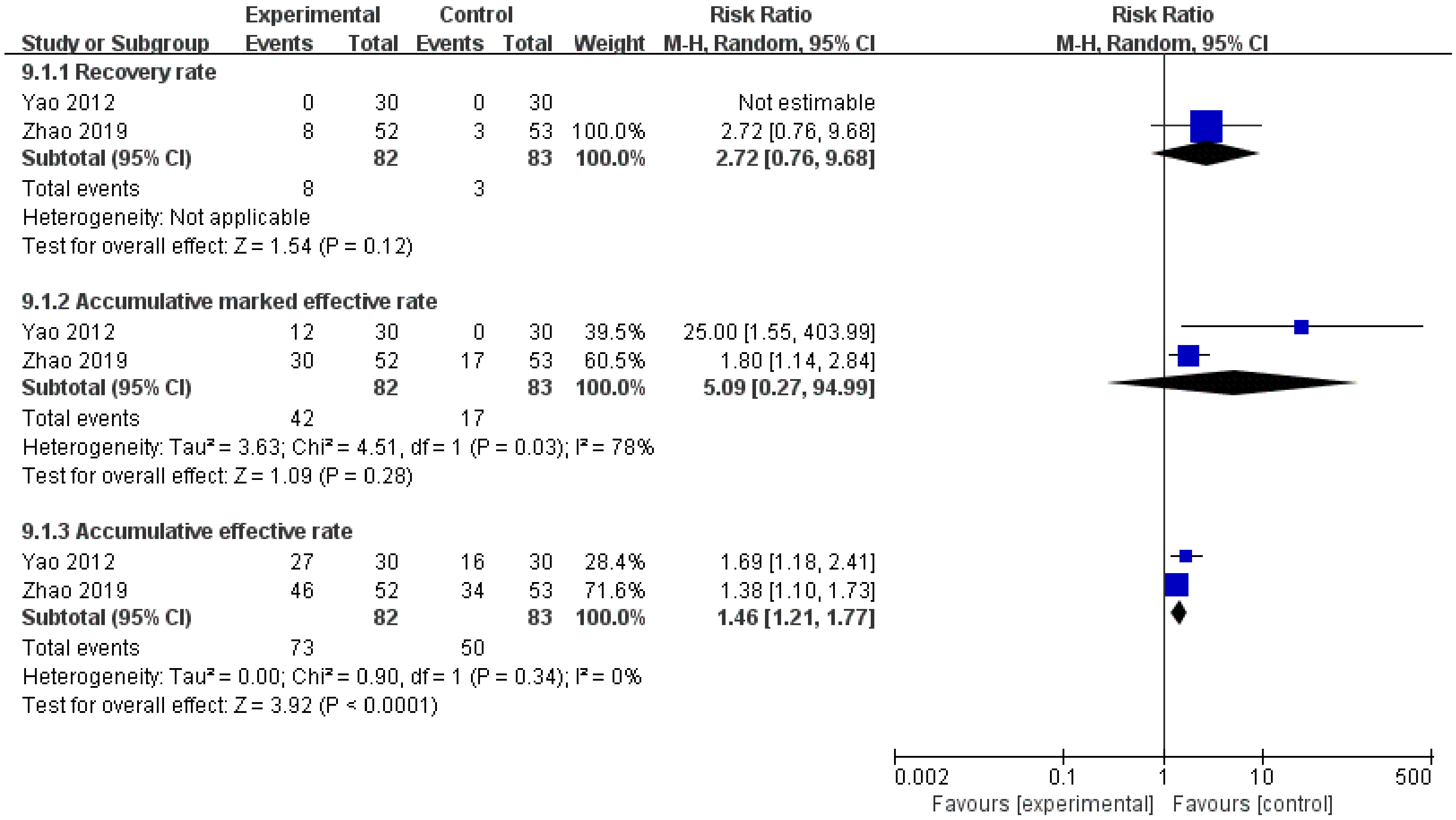

figure S12: Acupoint catgut embedding (ACE) plus oral Western medicine (OWM) versus OWM alone for diarrhea-predominant irritable bowel syndrome (IBS-D)
